# Update of: Kheang et al., Malaria Case Detection Among Mobile Populations and Migrant Workers in Myanmar: Comparison of 3 Service Delivery Approaches

**DOI:** 10.9745/GHSP-D-18-00358

**Published:** 2018-10-03

See updated article.

We have added French translations of the abstracts for a number of articles from the June 2018 issue (Volume 6, Number 2) in which the content focused on countries where the official language is French. This has affected the page numbering of these and subsequent articles. The new citations are as follows:

- Ouedraogo Y, Furlane G, Fruhauf T, et al. Expanding the single-visit approach for cervical cancer prevention: successes and lessons from Burkina Faso. Glob Health Sci Pract. 2018;6(2):288–298. https://doi.org/10.9745/GHSP-D-17-00326. *(French translation of the abstract added)*
- Harvey SA. Observe before you leap: why observation provides critical insights for formative research and intervention design that you'll never get from focus groups, Interviews, or KAP Surveys. Glob Health Sci Pract. 2018;6(2):299–316. https://doi.org/10.9745/GHSP-D-17-00328
- Koffi TB, Weidert K, Ouro Bitasse E, et al. Engaging men in family planning: perspectives from married men in Lomé, Togo. Glob Health Sci Pract. 2018;6(2):317–329. https://doi.org/10.9745/GHSP-D-17-00471. *(French translation of the abstract added)*
- Subramanian L, Simon C, Daniel EE. Increasing contraceptive use among young married couples in Bihar, India: evidence from a decade of implementation of the PRACHAR Project. Glob Health Sci Pract. 2018;6(2):330–344. https://doi.org/10.9745/GHSP-D-17-00440
- Sarma S, Nemser B, Cole-Lewis H, et al. Effectiveness of SMS technology on timely community health worker follow-up for childhood malnutrition: a retrospective cohort study in sub-Saharan Africa. Glob Health Sci Pract. 2018;6(2):345–355. https://doi.org/10.9745/GHSP-D-16-00290
- Marks KJ, Luthringer CL, Ruth LJ, et al. Review of grain fortification legislation, standards, and monitoring documents. Glob Health Sci Pract. 2018;6(2):356–371. https://doi.org/10.9745/GHSP-D-17-00427
- Ndiaye K, Portillo E, Ouedraogo D, Mobley A, Babalola S. High-risk advanced maternal age and high parity pregnancy: tackling a neglected need through formative research and action. Glob Health Sci Pract. 2018;6(2):372–383. https://doi.org/10.9745/GHSP-D-17-00417. *(French translation of the abstract added)*
- Kheang T, Lin MA, Lwin S, et al. Malaria case detection among mobile populations and migrant workers in Myanmar: comparison of 3 service delivery approaches. Glob Health Sci Pract. 2018;6(2):384–389. https://doi.org/10.9745/GHSP-D-17-00318
- Choi Y, Short Fabic M. Monitoring progress in equality for the Sustainable Development Goals: a case study of meeting demand for family planning. Glob Health Sci Pract. 2018;6(2):390–401. https://doi.org/10.9745/GHSP-D-18-00012

